# Carnivoran hunting style and phylogeny reflected in bony labyrinth morphometry

**DOI:** 10.1038/s41598-018-37106-4

**Published:** 2019-01-11

**Authors:** Julia A. Schwab, Jürgen Kriwet, Gerhard W. Weber, Cathrin Pfaff

**Affiliations:** 1 Department of Palaeontology, Faculty of Earth Science, Geography and Astronomy, University of Vienna, Geozentrum, Althanstraße 14, 1090 Vienna, Austria; 2 School of GeoSciences, Grant Institute, University of Edinburgh, The King’s Buildings, James Hutton Road, Edinburgh, EH9 3JW UK; 3 Department of Anthropology & Core Facility for Micro-Computed Tomography, Faculty of Life Science, University of Vienna, Althanstraße 14, 1090 Vienna, Austria

## Abstract

Carnivorans are a highly diverse and successful group of mammals, found on the top of the food chain. They originated in the Palaeocene (ca. 60 Ma) and have developed numerous lifestyles, locomotion modes and hunting strategies during their evolutionary history. Mechanosensory organs, such as the inner ear (which houses senses of equilibrium and hearing), represent informative anatomical systems to obtain insights into function, ecology and phylogeny of extant and extinct vertebrates. Using µCT scans, we examined bony labyrinths of a broad sample of various carnivoran species, to obtain new information about hunting behaviours of ancient carnivorans. Bony labyrinths were digitally reconstructed and measurements were taken directly from these 3D models. Principal component analyses generally separated various hunting strategies (pursuit, pounce, ambush and occasional), but also support their phylogenetic relationships (Canoidea vs. Feloidea). The height, width and length of all three semicircular canals show functional morphological adaptations, whereas the diameter of the canals, the height of the cochlea and particularly the angle between the lateral semicircular canal and the cochlea indicate a phylogenetic signal. The results demonstrate that the bony labyrinth provides a powerful ecological proxy reflecting both predatory habits as well as phylogenetic relationships in extinct and extant carnivorans.

## Introduction

Carnivorans are a highly diverse and well-documented group of mammalian top predators. They comprise two monophyletic clades, the Canoidea (dog–like carnivorans) and Feloidea (cat–like carnivorans)^1^ that can be distinguished based on morphological (e.g.^2^), and molecular data (e.g.^3^). However, the timing of the split between the two major clades has not been fully resolved^4,5^. These animals originated in the Palaeocene and distinctly diversified in the last 60 Million years^1,6,7^. During their evolutionary history, they have evolved to become morphologically disparate and have established an array of hunting strategies, behaviours and lifestyles, as well as a tremendous spatial range of habitats on almost all continents^8^. Comparatively large hunters pursue their prey over wide distances, whereas others are perfectly adapted to ambush or stalk their prey^8^. Pouncing on prey is more common in smaller carnivorans while specialised feeders on either fruits or insects rarely hunt at all^8^. However, determining if extinct carnivorans possessed similar variation in their hunting behaviours remains a challenge.

**Figure 1:**
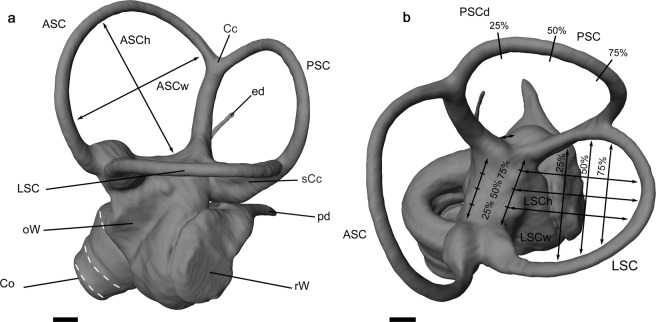
Left bony labyrinth of *Canis lupus* (Grey wolf). (**a**) lateral view, (**b**) dorsal view. ASC, anterior semicircular canal; ASCh, height of the anterior semicircular canal; ASCw, width of the anterior semicircular canal; Cc, crus commune; Co, cochlea; ed, endolymphatic duct; LSC, lateral semicircular canal; LSCh, height of the lateral semicircular canal; LSCw, width of the lateral semicircular canal; oW, oval window; pd, perilymphatic duct; PSC, posterior semicircular canal; PSCd; diameter of the posterior semicircular canal; rW, round window; sCc, secondary crus commune. Scale 1 mm.

Previous studies attempted to infer the predatory niches of extinct carnivorans using dental (e.g.^9–13^), and limb morphologies (e.g.^14–16^). More recently however, there has been an increase in studies using the inner ear, the sensory organ responsible for equilibrium and hearing as a proxy for inferring ecological niche (Fig. 1). The bony labyrinth encloses the membranous system (together know as inner ear), including three semicircular ducts (SCCs; ASC – anterior semicircular canal; PSC – posterior semicircular canal, LSC – lateral semicircular canal) for angular acceleration, and the utricle and saccule within the vestibule for linear acceleration^17^. Located in the petrosal bone it comprises one of the best-preserved anatomical systems in the fossil record. The organ of equilibrium (vestibular system) reflects orientation in three dimensional space and hence mirrors functional morphological aspects and enables insights into ecological habits (e.g.^18–30^). It has already been proven that more agile animals, performing faster head movements, developed larger semicircular canals to improve their body balance while moving in complex 3D environments (e.g.^23,30^), than those animals having a slower mode of locomotion (e.g.^18,20–28,31–33^). Therefore bony labyrinth morphometry represents a powerful methodology to correlate cranial sensory changes with functional patterns and hence represents a proxy for ecological adaptations. The cochlea (Co), however, is responsible for sound detection and shows major morphological differences, which might represent phylogenetic correlations within vertebrates (e.g.^33–36^).

Here, we use the most diversified sample of carnivoran bony labyrinths yet assembled to predict hunting behaviours in extinct taxa, providing novel insights into the evolution, lifestyle and especially hunting strategies of carnivoran mammals.

## Results

**Figure 2:**
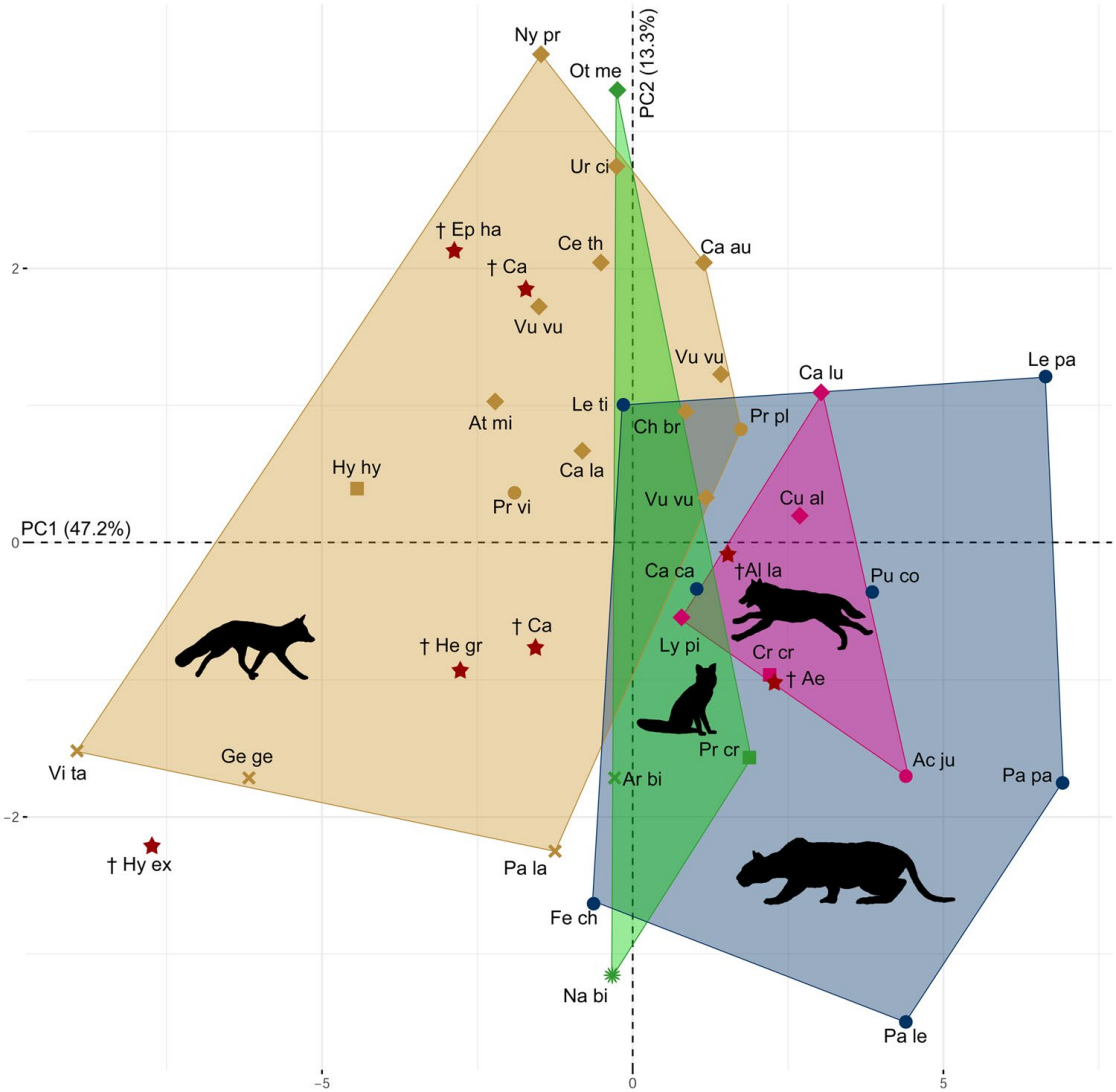
Principal component analysis (PCA) according to hunting strategies in carnivorans. Shape differences in the bony labyrinth. PC1 explains 47.2% of the variance; PC2 explains 13.3% of the variance. Coloured morphospace are defined as follows: brown: pounce; blue: ambush; pink: pursuit; darkgreen: occasional. Circles: Feloidea (Ac ju, *Acinonyx jubatus*; Ca ca, *Caracal caracal*; Fe ch, *Felis chaus*; Le pa, *Leopardus pardalis*; Le ti, *Leopardus tigrinus*; Pa le, *Panthera leo*; Pa pa, *Panthera pardus*; Pr pl, *Prionailurus planiceps*; Pr vi, *Prionailurus viverrinus*; Pu co, *Puma concolor*); squares: Hyaenidae (Cr cr, *Crocuta crocuta*; Hy hy, *Hyaena hyaena*; Pr cr, *Proteles cristatus*); cross: Viverridae (Ar bi, *Arctictis binturong*; Ge ge, *Genetta genetta*; Pa la, *Paguma larvata*; Vi ta, *Viverra tangalunga*); sun: Nandiniidae (Na bi, *Nandinia binotata*); diamonds: Canidae (At mi, *Atelocynus microtis*; Ca au, *Canis aureus*; Ca la, *Canis latrans*; Ca lu, *Canis lupus*; Ce th, *Cerdocyon thous*; Ch br, *Chrysocyon brachyurus*; Cu al, *Cuon alpinus*; Ly pi, *Lycaon pictus*; Ny pr, *Nyctereutes procyonoides*; Ot me, *Otocyon megalotis*; Ur ci, *Urocyon cinereoargenteus*; Vu vu, *Vulpes vulpes*); red/stars: extinct specimens (Ae, ^†^*Aelurodon* sp.; Al la, ^†^*Alopex lagopus*; Ca, ^†^*Canis* sp.; Ep ha, ^†^*Epicyon haydeni*; He gr, ^†^*Hesperocyon gregarius*; Hy ex, ^†^*Hyaenodon exiguus*).

**Figure 3:**
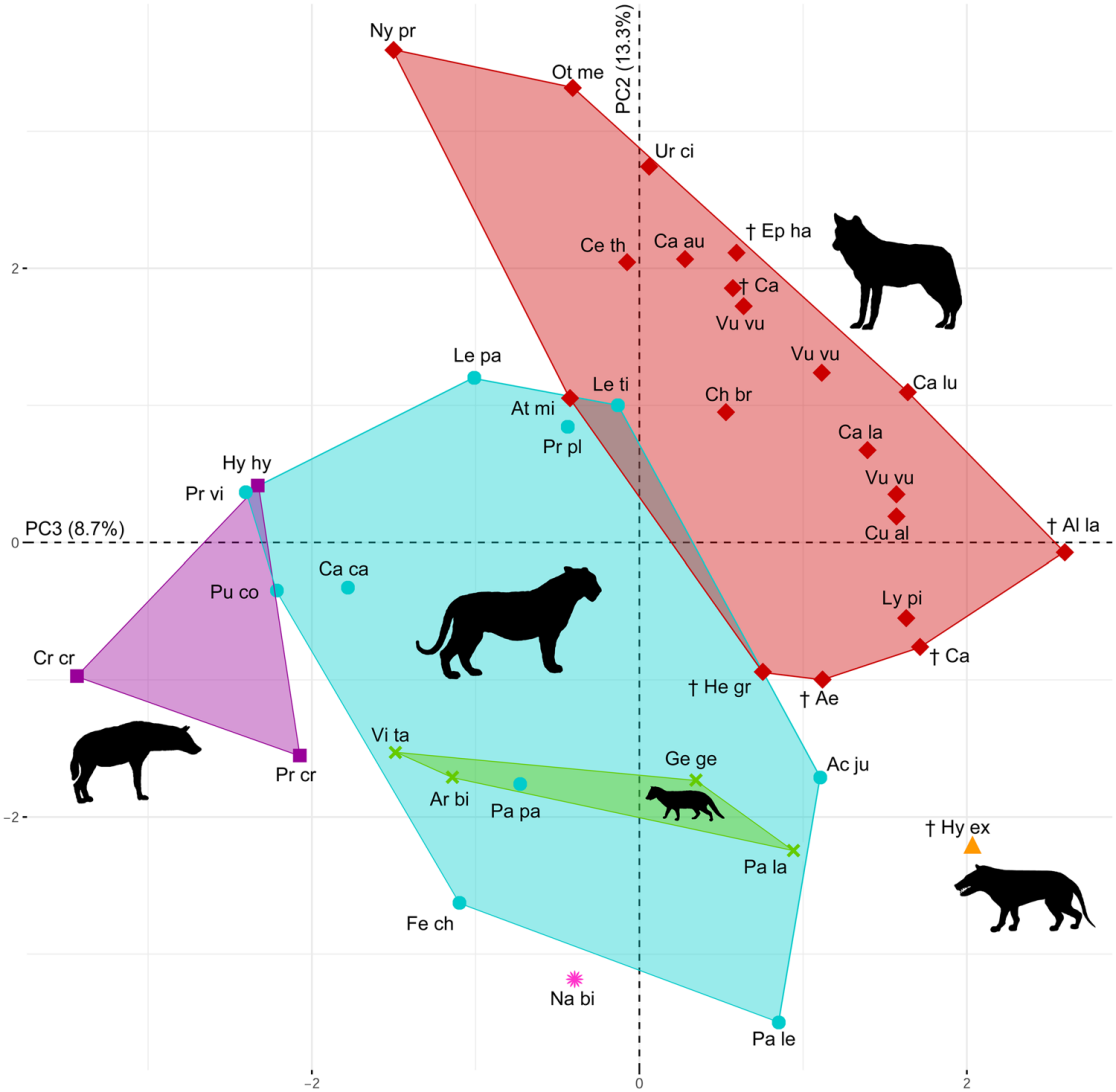
Principal component analysis (PCA) according to the family level of carnivorans. Shape differences in the bony labyrinth. PC2 explains 13.3% of the variance; PC3 explains 8.7% of the variance. Coloured morphospace are defined as follows: red/diamond: Canidae; cyan/circles: Felidae; purple/square: Hyaenidae; lightgreen/cross: Viverridae; pink/sun: Nandiniidae; orange/triangle: Hyaenodontidae. For data labels see Fig. 2.

The functional morphological and phylogenetic signal was statistically investigated using standardised bony labyrinth measurements (Supplementary Dataset 1) and additionally a principal component analysis (PCA; Figs 2 and 3). The first axes of the PCA explains 47.2%, the second axis 13.3% and the third PC axis 8.7% of the shape variation.

The first principal component (PC1) correlates positively with the height, width, length and radius of all three SCCs; the length of the crus commune (Cc); the length, width and height of the Co and the ASC/LSC, LSC/PSC and LSC/Co angles, but correlates negatively with the ASC/PSC angles. The second principal component (PC2) correlates positively with the width and diameter of the ASC; the length and diameter of the PSC and LSC; the length of the Cc; the height, width and length of the Co and the angles LSC/PSC and LSC/Co, but correlates negatively with the height, length and radius of the ASC; the height, width and radius of the PSC and LSC and the angles ASC/PSC and ASC/LSC. The third principal component (PC3) correlates positively with the width and length of the ASC; the height and length of the PSC; the width, radius and length of the LSC; the length of the Cc; the height of the Co and all measured angles (ASC/PSC, ASC/LSC, LSC/PSC, LSC/Co), but correlates negatively with the height, diameter and radius of the ASC; the height, diameter and radius of the PSC; the height and diameter of the LSC and the width and length of the Co.A detailed list of the PCA scores is found in the electronic supplementary material (Supplementary Dataset 1).

The results of our phylogenetic test reveal that the size of all three semicircular canals and additionally PC1 (linked with ecology) are not influenced by phylogenetic information (detailed values in Supplementary Dataset 1 and Data 3). The cochlea however, is influenced by phylogenetic traits in carnivorans. In the CVA the different hunting strategies observed in the investigated carnivorans separate well, as also seen in the PCA. To test the significance of the morphological differences we used a MANOVA. This indicates an overall classification accuracy of 82.05% for the hunting strategies and 91.89% based on the family level of the carnivorans.

## Discussion

Carnivorans have been a major research topic for decades (e.g.^2,3,37,38^). However, studies on their vestibular system have only recently come into focus, but are still restricted to specific groups (e.g. Feloidea^28,30^; Musteloidea^27^; *Canis*^39^). Nevertheless, it has been demonstrated that the vestibular system, representing the organ of equilibrium, provides a powerful proxy for reconstructing ecological preferences in vertebrates (e.g.^18–20,22–24,26–28,30–33,40–45^). Differences in ecology are related to morphological changes of the inner ear, particularly of the three semicircular canals, as they provide information for angular acceleration necessary to balance the body in complex 3D environments. During locomotion and predation, it is of major importance to stabilise the head and gaze (vestibulo-ocular and vestibulo-collic reflexes) especially for fast moving species^46^. In cetaceans, however, the cochlea can be correlated with habitat preferences, due to association with different echolocation abilities in differing habitats and additionally size is correlated with environment^36^.

Here, we investigated the bony labyrinth morphometry as a proxy for hunting behaviours and predatory niche adaptations in extinct carnivorans and additionally gain a better understanding of their evolutionary history. Correlations between size of the semicircular canals and hunting behaviour and speed are identified and visually shown in the PCA (Fig. 2). Previous studies demonstrate that high-speed hunting in cheetahs can be correlated with an enlargement of the vestibular system and elongation of the ASC and PSC^30^ and similarly, feloideans exhibit ecological bony labyrinth adaptations in the size of their semicircular canals^28^. We recognised distinct morphospaces, based on PC1, showing the highest loadings on the relative height, width and length of all three semicircular canals (Fig. 2). Four different hunting behaviours are present in the examined taxa (pounce, pursuit, ambush, occasional; defined after^47^, Table 1) showing an overall classification accuracy of 82% (Supplementary Data 2). Hence, carnivoran hunting behaviour is reflected in the size of the semicircular canals. A clear distinction is present between the hunting styles pounce and pursuit, and pounce and ambush, respectively. The range of the pounce and ambush morphospaces is much larger than of the pursuit and the occasional ones. Those species exhibiting an occasional diet and hence rarely hunt at all, are placed in between, overlapping all of the other hunting styles. Additionally, major distinctions are present in the overall hunting behaviour of feloids and canids. Generally, feloids are solitary, ambush hunters, able to retract their claws and developed more flexible forelimbs during their evolution to grapple with and hold their prey^8,48,49^. Canids, conversely, lack these features. Large canids, as the African hunting dog (*Lycaon pictus*) or the grey wolf (*Canis lupus*), need to organise in groups when hunting on large prey^8^. This is reflected in the morphospace reconstructions where they are assigned to the pursuit, comparative hunter niche^48^. However, it is assumed that an ambush hunting style is observed in large Borophaginae (an extinct canid subfamily), such as *Epicyon haydeni*^14^. Based on their strong teeth and robust skull^50^, most large extinct canids might have had a scavenging lifestyle and are known as ‘hyaenoid dogs’^13^. However, recent studies suppose a combined and unique predation strategy for fossil canids^15^. Viverridae and Nandiniidae however, are not that active and fast hunters as canids and feloids are, they generally have a solidary and omnivorous lifestyle^8^. Hyaenidae show the most variable hunting behaviour within carnivorans, the striped hyena (*Hyaena hyaena*) is a scavenger, the spotted hyena (*Crocuta crocuta*) is generally an active pack hunter and the Aardwolf (*Proteles cristatus*) is insectivore and specialised on termites^8^. This coincide with the PCA, as all three species plot in the respective morphospace. Here, ambush and pursuit predation as faster hunting strategies are reflected in larger semicircular canals represented in the relative height, width and length. This coincides with other carnivoran studies^30,44^, but contrasts with the functional morphological signal found in the diameter of the canals in the squirrel-related clade and marsupials^26,28^. However, based on carnivoran bony labyrinth morphometry our results clearly demonstrate that the extinct *Hesperocyon gregarius*, *Epicyon haydeni*, *Canis* sp., *Alopex lagopus* and *Hyaenodon exiguus* exhibited a pounce predation, whereas *Aelurodon* sp. developed a pursuit hunting strategy.

**Table 1:** Extant specimens. Including five different carnivoran families: Canidae, Viverridae, Hyaenidae, Nandiniidae, Felidae.

Family	Species	Common name	Hunting style
Canidae	*Canis lupus*	Grey wolf	pursuit
Canidae	*Atelocynus microtis*	Short eared dog	pounce
Canidae	*Cerdocyon thous*	Crab eating fox	pounce
Canidae	*Chrysocyon brachyurus*	Maned wolf	pounce
Canidae	*Cuon alpinus*	Dhole	pursuit
Canidae	*Lycaon pictus*	African wild dog	pursuit
Canidae	*Nyctereutes procyonoides*	Racoon dog	pounce
Canidae	*Otocyon megalotis*	Bat eared fox	occasional
Canidae	*Urocyon cinereoargenteus*	Grey fox	pounce
Canidae	*Vulpes vulpes*	Red fox	pounce
Canidae	*Canis latrans*	Coyote	pounce
Canidae	*Canis aureus*	Golden jackal	pounce
Felidae	*Felis chaus*	Jungle cat	ambush
Felidae	*Panthera leo*	Lion	ambush
Felidae	*Acinonyx jubatus*	Cheetah	pursuit
Felidae	*Panthera pardus*	Leopard	ambush
Felidae	*Leopardus pardalis*	Ocelot	ambush
Felidae	*Leopardus tigrinus*	Oncilla	pounce
Felidae	*Caracal caracal*	Caracal	ambush
Felidae	*Puma concolor*	Puma	ambush
Felidae	*Prionailurus planiceps*	Flat-headed cat	pounce
Felidae	*Prionailurus viverrinus*	Fishing cat	pounce
Hyaenidae	*Hyaena hyaena*	Striped hyena	pounce
Hyaenidae	*Proteles cristatus*	Aardwolf	occasional
Hyaenidae	*Crocuta crocuta*	Spotted hyena	pursuit
Nandiniidae	*Nandinia binotata*	African palm civet	occasional
Viverridae	*Arctictis binturong*	Binturong	occasional
Viverridae	*Viverra tangalunga*	Malay civet	pounce
Viverridae	*Genetta genetta*	Common genet	pounce
Viverridae	*Paguma larvata*	Masked palm civet	pounce

**Figure 4:**
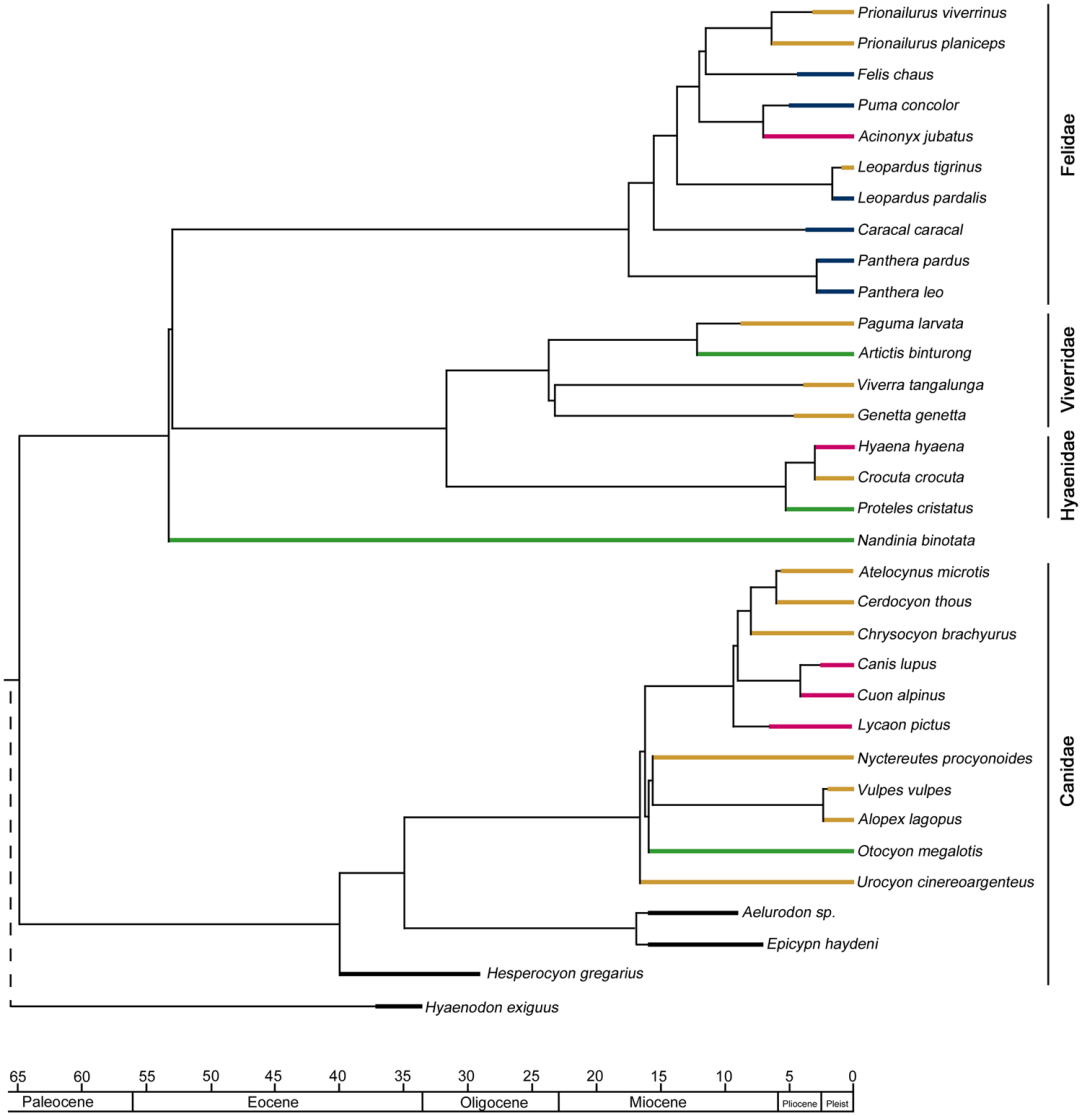
Phylogeny of the examined specimens^3^. Colours represent the respective hunting behaviour: brown: pounce; blue: ambush; pink: pursuit; darkgreen: occasional.

The vestibular system, however, not only reflects ecological adaptations but also reveals information about phylogenetic relationships between the Canoidea and Feloidea (Fig. 4). Four carnivoran families (Hyaenidae, Viverridae, Nandiniidae and Canidae) can be clearly distinguished from each other (Fig. 3), and only the diverse Felidae show minor overlapping regions with other taxa. The extinct *Hyaenodon exiguus* fits in none of the defined phylogenetic morphospaces and has an intermediate position, which supports its position outside of the carnivorans^51^. Our results demonstrate that the most important trait bearing a phylogenetic signal in carnivorans is the bias angle^26^ between the LSC and the cochlea, and additionally the diameter of all three semicircular canals and the height of the cochlea, as it already was postulated for several feloidean families^44^. Canidae unambiguously developed a larger angle between LSC/Coc and additionally show a larger cochlea height compared to feloideans. However, the presence of a phylogenetic signal contrasts with investigations of both the squirrel-related clade (fossorial vs. arboreal^26^), as well as marsupials (saltorial vs. arboreal^28^), which show a functional morphological rather than a phylogenetic adaptation in the diameter of the SCCs. Additionally, the entire bony labyrinth shape infers phylogenetic relationships in various other mammalian families, such as Musteloidea, ruminants or cetaceans^27,33,36,41^.

In conclusion, the bony labyrinth morphometry clearly provides both a strong functional morphological signal as correlation with varying predation strategies, as well as a phylogenetic signal in carnivorans. Overall, the size of all three bony labyrinth semicircular canals have altered during the evolutionary history of carnivorans to adapt to different hunting strategies, with fast predators showing larger SCCs. Furthermore, the angle between the LSC and the cochlea, the diameter and the height of the cochlea contain phylogenetic information. Thus, the bony labyrinth morphology is a useful proxy for estimating predatory adaptations within a phylogenetic context in extinct carnivoran mammals.

## Methods

Bony labyrinths of 36 specimens of five different carnivoran families and *Hyaenodon exiguus* (Tables 1 and 2) were used to estimate changes in hunting style during carnivoran evolutionary history, and additionally, to test for a phylogenetic signal. New data of canid specimens were combined with published measurements of feloideans^44^. Most specimens are housed in the collection of the Natural History Museum in Vienna (NHMW) and the collection of the Department of Palaeontology at the University of Vienna (IPUW), additional extinct canids are housed at the Field Museum of Natural History in Chicago (FMNH; *Aelurodon* sp., *Hesperocyon gregarius*), the American Museum of Natural History in New York (AMNH; *Epicyon haydeni*).

**Table 2:** Extinct specimens. Including Canidae with three different subfamilies and Hyaenodontidae.

Family	Subfamily	Species	Timeperiod
Canidae	Hesperocyoninae	^†^ *Hesperocyon gregarius*	40–29 Ma
Canidae	Borophaginae	^†^ *Epicyon haydeni*	16–7 Ma
Canidae	Borophaginae	^†^*Aelurodon* sp.	16–9 Ma
Canidae	Caninae	^†^*Canis* sp.	6 Ma - recent
Canidae	Caninae	^†^ *Alopex lagopus*	2.5 Ma - recent
Hyaenodontidae	Hyaenodontidae	^†^ *Hyaenodon exiguus*	37.2–33.8 Ma

The skulls of the specimens were scanned non-invasively using µCT devices. The majority of the specimens were scanned at the Department of Palaeontology of the University of Vienna (SkyScan/Bruker 1173), the skulls of *Canis lupus* and *Epicyon haydeni* were scanned at the Department of Anthropology at the University of Vienna (Viscom X8060) and two fossil specimens (*Hesperocyon gregarius* and *Aelurodon* sp.) were scanned at the Department of Organismal Biology and Anatomy at the PaleoCT Luo Lab (GE v|tome|x scanner) at the University of Chicago. A detailed list of scanning settings is found in the electronic supplementary material (Supplementary Dataset 1).

Scanning images were visualised, bony labyrinths were segmented manually and virtually reconstructed three dimensionally using the software Amira 5.4.5 (Visualization Sciences Group). For comparison of morphological traits, only left labyrinths were reconstructed. Measurements were taken directly on the 3D labyrinths following the protocol of previous studies (Fig. 1)^19,26,28,44,52^. All measurements were scaled in millimetre related to the voxel (three-dimensional pixel) size. A detailed list of the 3D measurements is found in the electronic supplementary material (Supplementary Dataset 1).

Statistical analyses of the anatomy of the bony labyrinth were performed, using the software R version 1.0.136^53^. First, the mean of the measurements of the length, width and diameter of each canal was calculated. Additionally, linear regression and residuals of the measurements and the condylobasal length (CBL) were calculated for standardisation and to create size independent values^26,28,44^. A Principal Component Analysis (PCA; Figs 2 and 3) was performed and delimitable morphospaces were defined using the R packages FactoMineR^54^ and factoextra^55^, to correlate bony labyrinth morphology and hunting strategies as well as phylogenetic signals. A Canonical Variate Analysis (CVA; Supplementary Data 1) was calculated for the values of the PC axis and furthermore the Multivariate Analysis of Variance (MANOVA; Supplementary Data 2) using Morpho 2.6^56^. A phylogenetic tree has been superimposed on the PCA using the R package phytools^57^ (Supplementary Data 5). Additionally, the phylogenetic influences in the vestibular system was calculated using the phylogenetic independent contrast (PIC), Blomberg’s K value and the Pagel’s lambda^58–60^ using Mesquite version 3.2, R 1.0.136^53^ and the R – packages ape 4.1^61^, phylobase 0.8.2^62^ and phylotools 0.6-00^57^. Phylogenetic analyses are based on the carnivoran supertree^3^. A detailed list of the calculated phylogenetic values is found in the electronic supplementary material (Supplementary Dataset 1).

The anatomical variance of the semicircular canals of the bony labyrinth was calculated for all specimens of the species *Vulpes vulpes* and additionally for all specimens of the genus *Canis* using the ‘coefficient of variability’^63^ (Supplementary Data 4).

## Supplementary information

Supplementary Dataset 1

Supplementary Dataset 2

## Data Availability

All data generated and analysed during this study are given in Supplementary data.
