# Supplementary material for: Carnivoran hunting style and phylogeny reflected in bony labyrinth morphometry
Source: Sci Rep. 2019 Jan 11;9:70. doi: 10.1038/s41598-018-37106-4 (PMC6329752; doi:10.1038/s41598-018-37106-4)

Carnivoran hunting style and phylogeny reflected in bony labyrinth morphometry

Julia A. Schwab, Jürgen Kriwet, Gerhard W. Weber, Cathrin Pfaff

<u>Supplementary Data 1</u>: CVAs on the PC values.

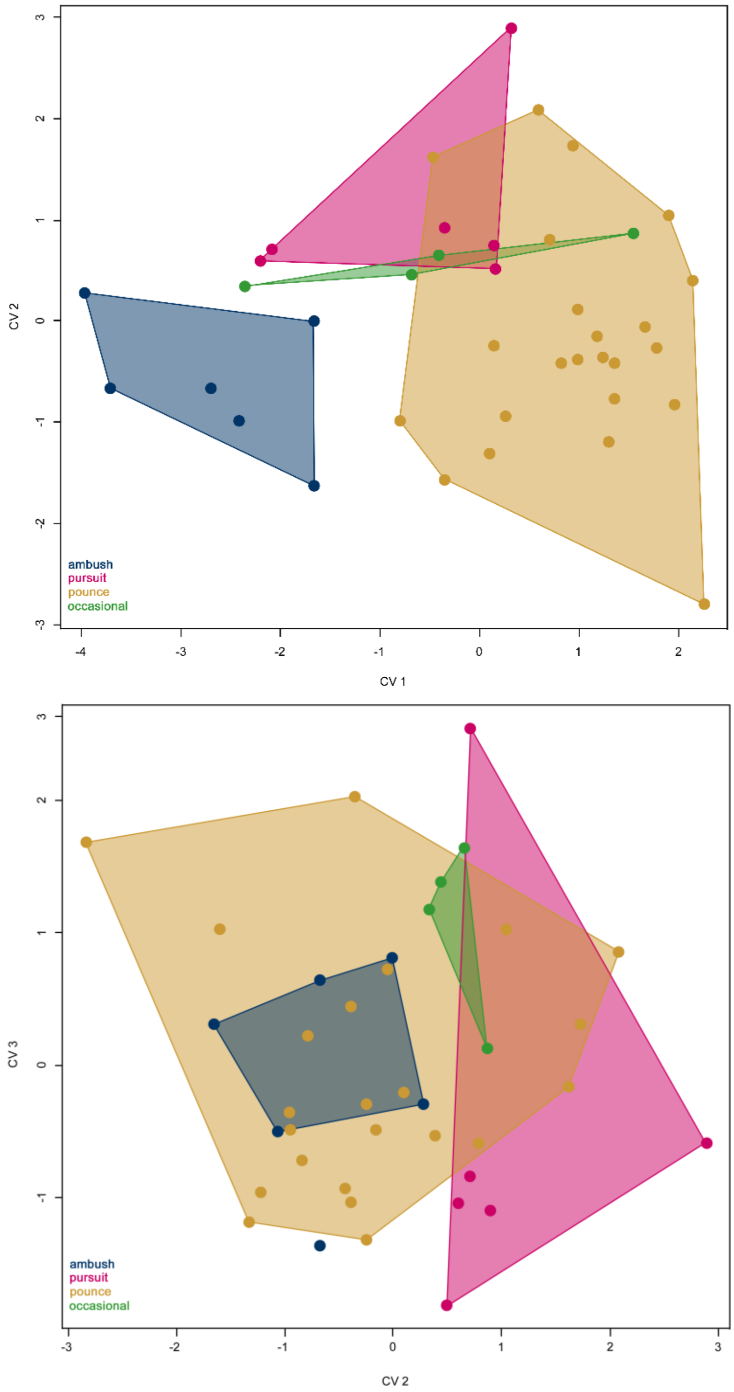

		CV 1	CV 2	CV 3
At mi	pounce	1.330574906	-0.396995278	0.448622598
Ce th	pounce	0.974994161	0.101655861	-0.217717598
Ch br	pounce	0.939269896	1.712101662	0.304635336
Cu al	pursuit	-0.348886915	0.897668105	-1.102860918
Ly pi	pursuit	0.140064507	0.736803525	-0.827600278
Ny pr	pounce	1.680299875	-0.037016869	0.711594539
Ot me	occasional	1.537956004	0.856117571	0.115415029
Ur ci	pounce	1.203325576	-0.139412646	-0.487984413
Ep ha	pounce	1.961324327	-0.842643381	-0.714045489
Ae	pounce	0.591772105	2.091802972	0.85982975
He gr	pounce	1.920954898	1.03721255	1.029605618
Al la	pursuit	0.160584334	0.519696701	-1.817168065
Vu vu	pounce	0.878284216	0.102796487	-0.734671417
Ca lu	pounce	0.706026265	-0.089941741	-0.947896158
Ac ju	pursuit	-2.207191635	0.595131744	-1.035802715
Ar bi	occasional	-0.408591954	0.672630935	1.652515007
Cr cr	pursuit	-2.070063886	0.72406446	2.538996464
Fe ch	ambush	-1.648614049	-0.015850319	0.784575118
Ge ge	pounce	1.357145703	-0.781853615	0.22723674
Hy hy	pounce	1.228331526	-0.371343849	2.029049541
Hy ex	pounce	2.138172263	0.416602293	-0.54224513
Le pa	ambush	-2.42196053	-1.012996778	-0.500731514
Le ti	pounce	0.253372023	-0.965711009	-0.380988344
Ly ca	ambush	-1.673909798	-1.635402367	0.311553995
Na bi	occasional	-0.699263021	0.445253916	1.380452943
Pa la	pounce	-0.486475561	1.63317986	-0.159412864
Pa le	ambush	-3.696128935	-0.676751811	-1.389369925
Pa pa	ambush	-3.955027599	0.28963317	-0.289273601
Pr pl	pounce	-0.789952301	-0.984024844	-0.496391412
Pr vi	pounce	-0.362058695	-1.564738956	1.029564234
Pr cr	occasional	-2.351453818	0.346285904	1.176635487
Pu co	ambush	-2.704727379	-0.69376361	0.639504654
Vi ta	pounce	2.241179998	-2.816016653	1.665300251

Supplementary Data 2: Multivariate analysis of Variance (MANOVA)

Hunting strategies

classification result in frequencies

	ambush	occasional	pounce	pursuit
ambush	6	0	0	0
occasional	1	2	1	0
pounce	0	0	22	1
pursuit	1	1	2	2

classification result in %

	ambush	occasional	pounce	pursuit
ambush	100	0	0	0
occasional	25	50	25	0
pounce	0	0	95.65	4.35
pursuit	16.6667	16.6667	33.33	33.33

overall classification accuracy: 82.05128 %

Kappa statistic: 0.68548

Family level

classification result in frequencies

	Canidae	Felidae	Hyaenidae	Viverridae	Nandiniidae	*Hyaenodon exiguus*
Canidae	20	0	0	0	0	0
Felidae	1	8	1	0	0	0
Hyaenidae	0	0	2	1	0	0
Viverridae	0	0	0	4	0	0
Nandiniidae	0	0	0	0	1	0
Hyaenodon exiguus	0	0	0	0	0	1

classification result in %

	Canidae	Felidae	Hyaenidae	Viverridae	Nandiniidae	*Hyaenodon exiguus*
Canidae	100	0	0	0	0	0
Felidae	10	80	10	0	0	0
Hyaenidae	0	0	66.667	33.333	0	0
Viverridae	0	0	0	100	0	0
Nandiniidae	0	0	0	0	100	0
Hyaenodon exiguus	0	0	0	0	0	100

overall classification accuracy: 91.89189 %

Kappa statistic: 0.86786

Supplementary Data 3: Blomberg's K value on the PC axis

	K - value	p - value
PC1	0.2786267	0.612
PC2	0.5027765	0.159
PC3	0.3654904	0.368

Supplementary Data 4: The variation of the three semicircular canals using the 'coefficient of variability' (Ekdale and Rowe, 2011) of *Vulpes vulpes* and all 5 specimen of the genus *Canis*

	Variability % ASC	Variability % PSC	Variability % LSC
Vulpes vulpes	6.12291	9.76443	4.53776
Canis	5.07469	5.32974	8.65412

Supplementary Data 5: superimposed phylogenetic tree on PCA

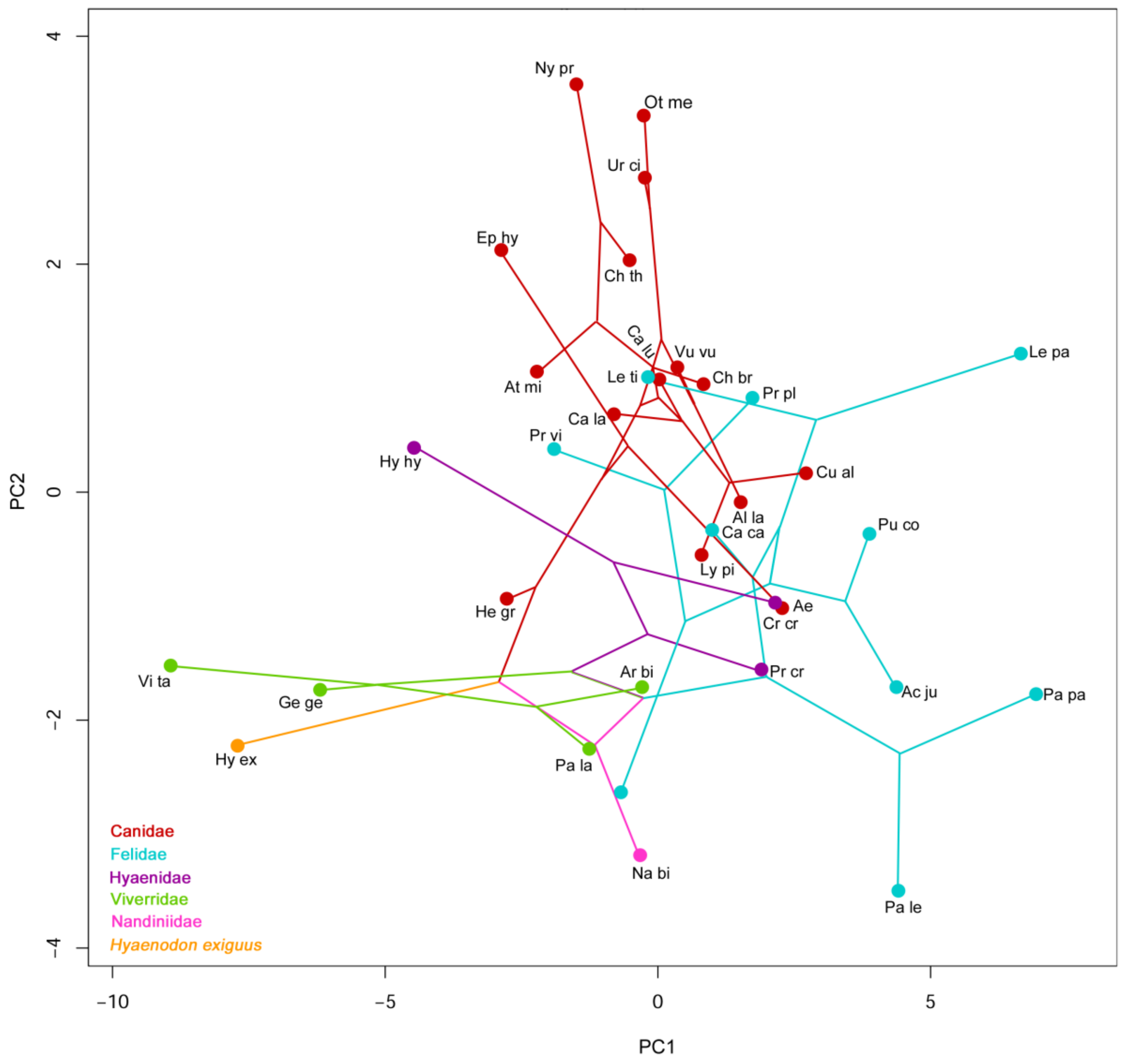

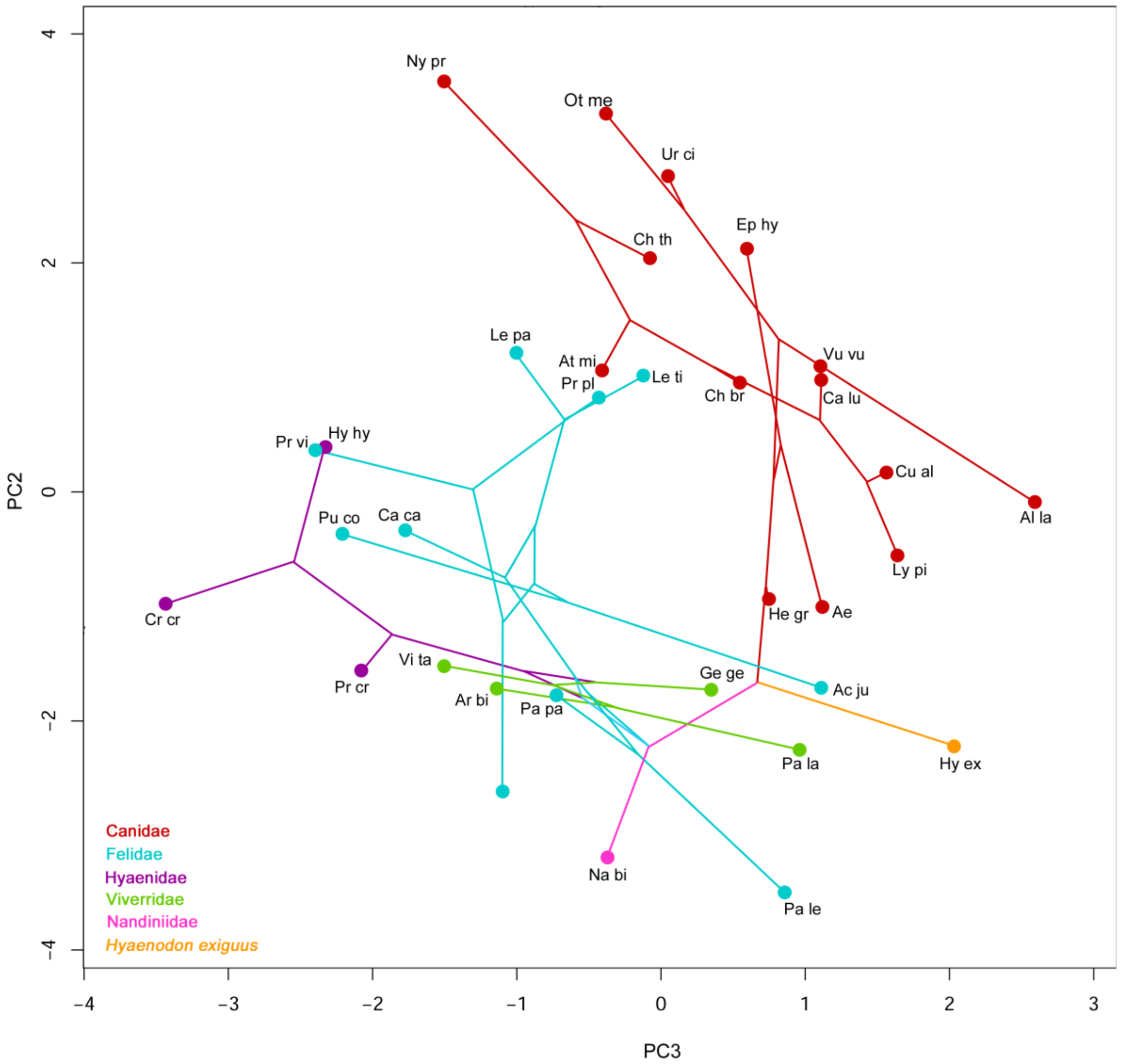
Ny pr
Ot me
Ur ci
Ep hy
Ch th
Le pa
At mi
Pr pl
Le ti
Ch br
Vu vu
Ca lu
Pr vi
Hy hy
Cu al
Al la
Pu co
Ca ca
Ly pi
Cr cr
He gr
Ae
Vi ta
Pr cr
Ge ge
Ar bi
Pa pa
Ac ju
Pa la
Hy ex
Na bi
Pa le
Canidae
Felidae
Hyaenidae
Viverridae
Nandiniidae
Hyaenodon exiguus
PC2
PC3
4
2
0
−2
−4
−4
−3
−2
−1
0
1
2
3

Supplementary Data 6: PCA without *Hyaenodon exiguus*

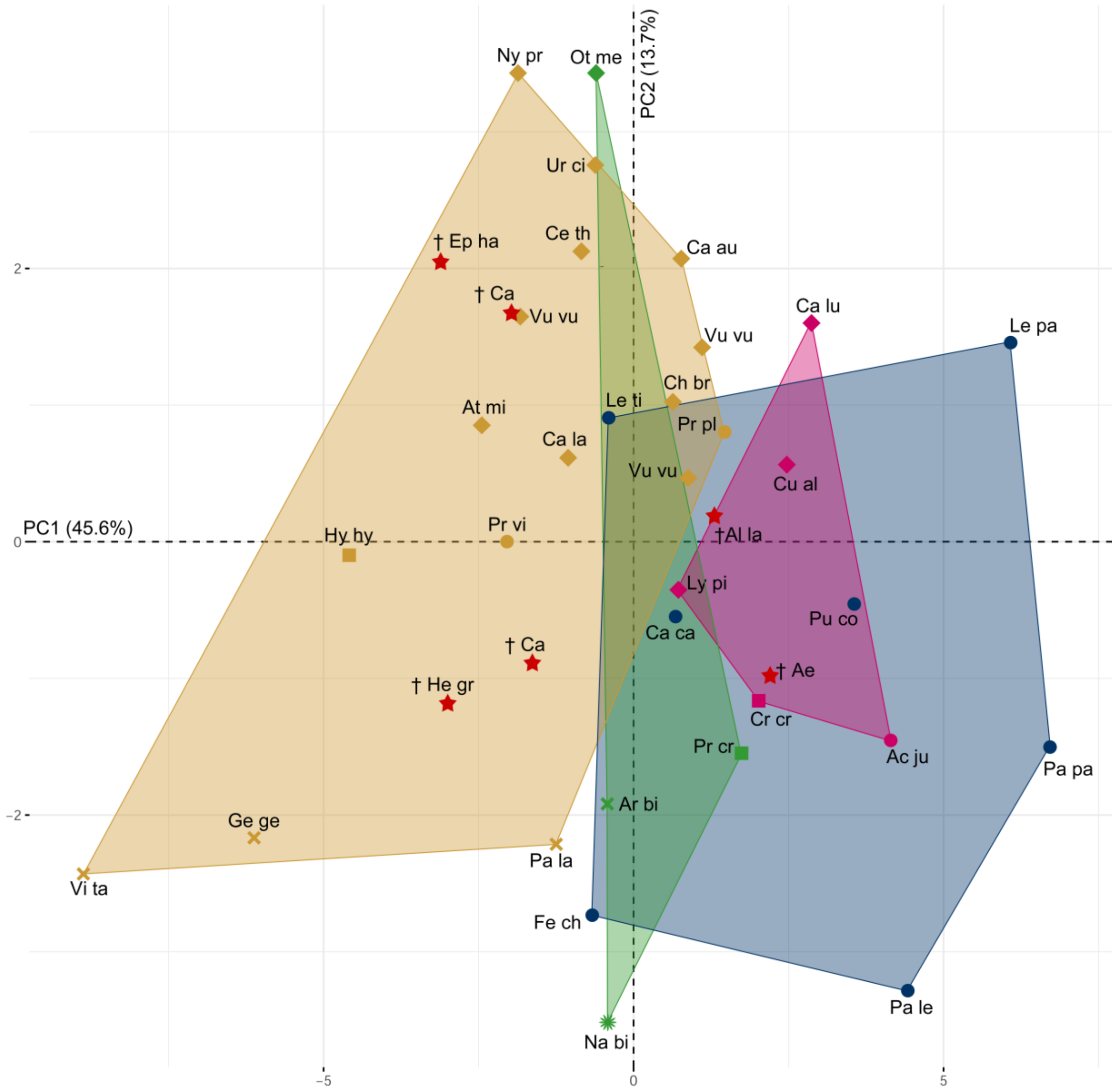

	PC1	PC2
ASCh	0.824422449	-0.188797545
ASCw	0.88210708	0.052468843
ASCd	0.287059038	0.793446061
ASCr	0.93848279	-0.072151018
ASCl	0.715361365	-0.031993509
PSCh	0.923614723	-0.192467935
PSCw	0.897091634	-0.125966074
PSCd	0.347579089	0.763899112
PSCr	0.93835258	-0.166588469
PSCl	0.822788395	0.207284013
LSCh	0.839566784	-0.330157331
LSCw	0.869225297	-0.061503013
LSCd	0.181958873	0.619559816
LSCr	0.905908938	-0.215944916
LSCl	0.638079686	0.313832806
LCc	0.636090965	0.124653282
Coch	0.073330299	0.792369182
Cocw	0.755887066	-0.002270155
Cocl	0.609795152	0.149454469
angl.ASC.PSC	0.062922763	-0.176043329
angl.ASC.LSC	0.354123698	-0.18075904
angl.LSC.PSC	0.098761981	0.263532304
angl.LSC.Co	0.032301806	0.580680784

Supplementary Data 7: CT slices of the fossil canid specimen.

Epicyon haydeni FM 61501 (67µm)

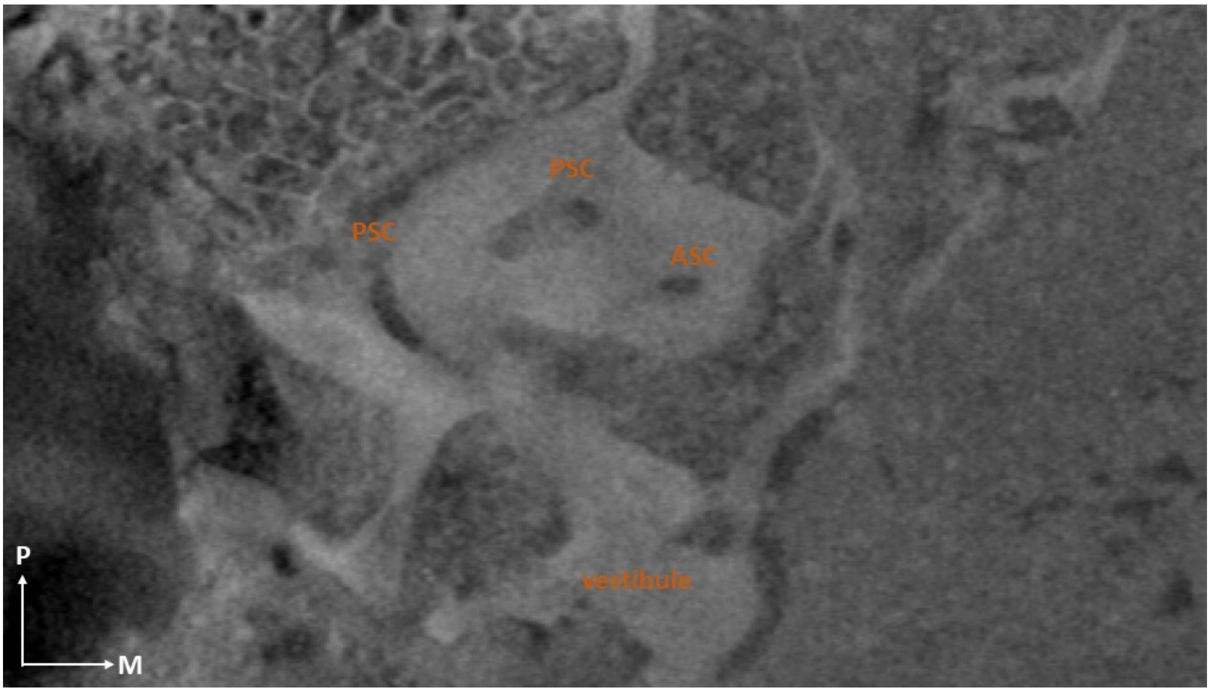

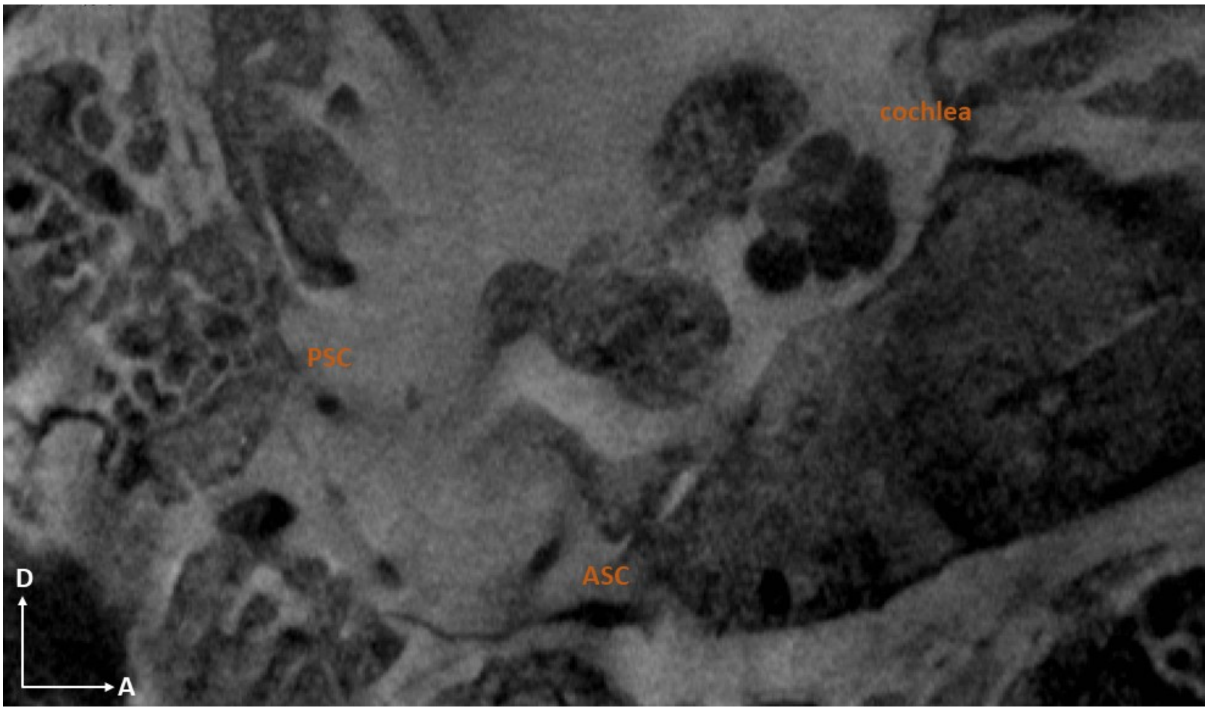

Aelurodon sp. FMNH P26186 (85.698μm)

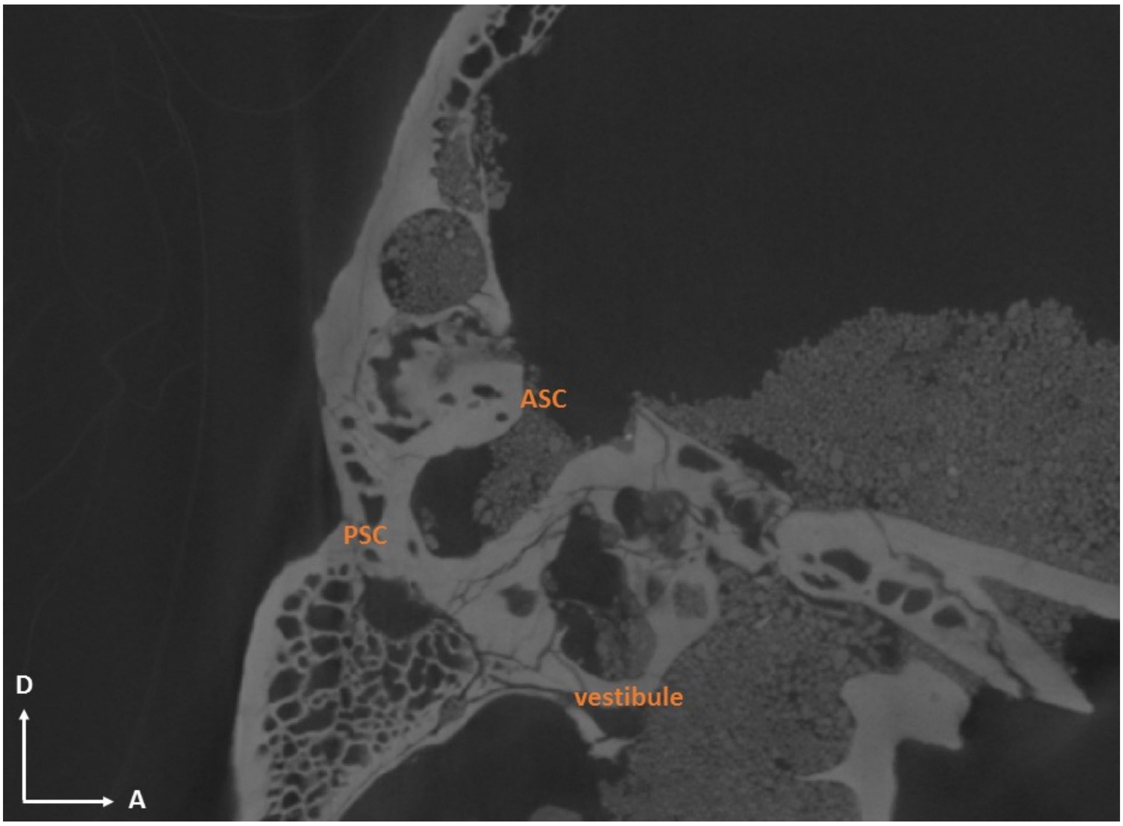

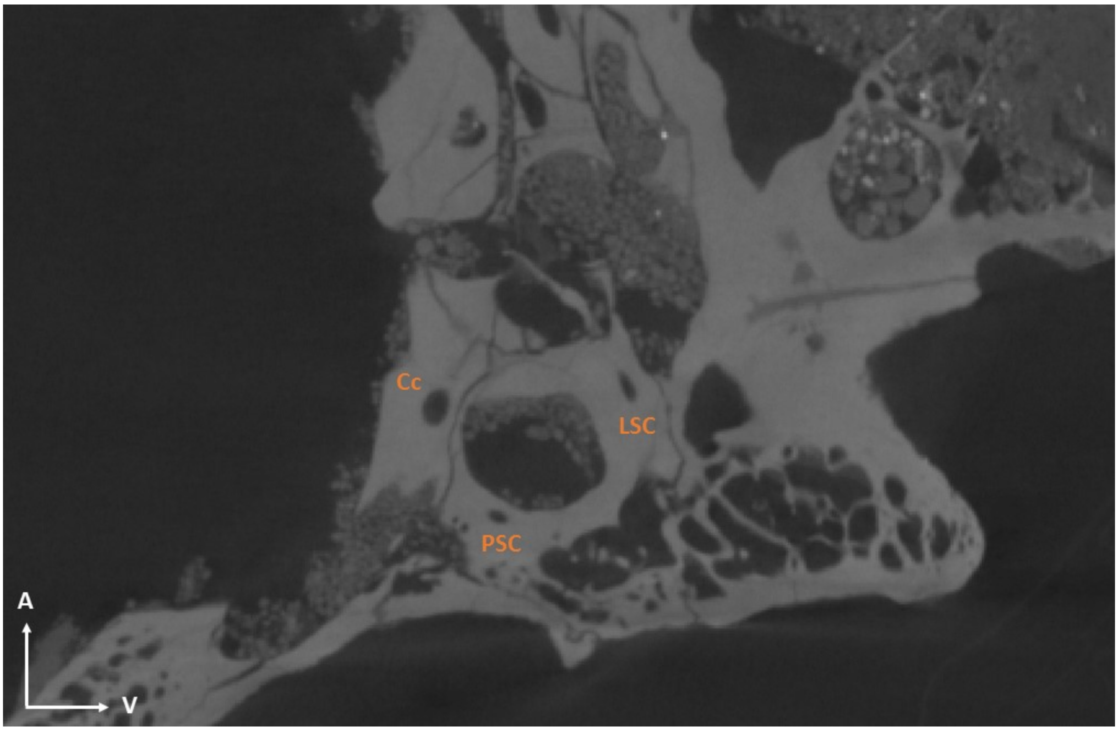

Supplement: Supplementary file 2 — Supplementary Dataset 2 [file 41598_2018_37106_MOESM2_ESM.pdf]
